# *Ganodermaovisporum* sp. nov. (Polyporales, Polyporaceae) from Southwest China

**DOI:** 10.3897/BDJ.10.e80034

**Published:** 2022-07-06

**Authors:** Hong-De Yang, Yong Ding, Ting-Chi Wen, Kalani Kanchana Hapuarachchi, De-Ping Wei

**Affiliations:** 1 Key Laboratory of Forest Biotechnology in Yunnan, Southwest Forestry University, Kunming, China Key Laboratory of Forest Biotechnology in Yunnan, Southwest Forestry University Kunming China; 2 The Engineering Research Center of Southwest Bio–Pharmaceutical Resources Ministry of Education, Guizhou University, Guiyang, China The Engineering Research Center of Southwest Bio–Pharmaceutical Resources Ministry of Education, Guizhou University Guiyang China; 3 Center of Excellence in Fungal Research, Mae Fah Luang University, Chiang Rai, Thailand Center of Excellence in Fungal Research, Mae Fah Luang University Chiang Rai Thailand; 4 State Key Laboratory Breeding Base of Green Pesticide and Agricultural Bioengineering, Guiyang, China State Key Laboratory Breeding Base of Green Pesticide and Agricultural Bioengineering Guiyang China; 5 The Mushroom Research Centre, Guizhou University, Guiyang, China The Mushroom Research Centre, Guizhou University Guiyang China; 6 State Key Laboratory Breeding Base of Green Pesticide and Agricultural Bioengineering, Key Laboratory of Green Pesticide and Agricultural Bioengineering, Ministry of Education, Guizhou University, Guiyang, China State Key Laboratory Breeding Base of Green Pesticide and Agricultural Bioengineering, Key Laboratory of Green Pesticide and Agricultural Bioengineering, Ministry of Education, Guizhou University Guiyang China; 7 Department of Entomology and Plant Pathology, Faculty of Agriculture, Chiang Mai University, Chiang Mai, Thailand Department of Entomology and Plant Pathology, Faculty of Agriculture, Chiang Mai University Chiang Mai Thailand

**Keywords:** one new species, *Ganoderma*, morphology, phylogeny, taxonomy

## Abstract

**Background:**

*Ganoderma* is a white-rot fungus with a cosmopolitan distribution and includes several economically important species. This genus has been extensively researched due to its beneficial medicinal properties and chemical constituents with potential nutritional and therapeutic values. Traditionally, species of *Ganoderma* were identified solely based on morphology; however, recent molecular studies revealed that many morphology-based species are conspecific. Furthermore, some type species are in poor condition, which hinders us from re-examining their taxonomic characteristics and obtaining their molecular data. Therefore, new species and fresh collections with multigene sequences are needed to fill the loopholes and to understand the biological classification system of *Ganoderma*.

**New information:**

In a survey of *Ganoderma* in Guizhou Province, southwest China, we found a new species growing on soil and, herein, it was identified by both morphology and phylogenetic evidence. Hence, we propose a new species, *Ganodermaovisporum* sp. nov. This species is characterised by an annual, stipitate, laccate basidiome, with a red–brown to brownish-black pileus surface and pale white pores, duplex context, clavate pileipellis terminal cells, trimitic hyphal system, ellipsoid basidiospores with dark brown eusporium bearing coarse echinulae and an obtuse turgid appendix. Phylogenetic analyses confirmed that the novel species sisters to *G.sandunense* with high bootstrap support. Furthermore, the RPB2 sequence of *G.sandunense* is supplied for the first time. Notably, we re-examined the type specimen of *G.sandunense* and provide a more precise description of the duplex context, pileipellis terminal cells and basidia. All species collected are described and illustrated with coloured photographs. Moreover, we present an updated phylogeny for *Ganoderma*, based on nLSU, ITS, RPB2 and TEF1-α DNA sequence data and species relationships and classification are discussed.

## Introduction

Ganodermataceae Donk is a large family of Polyporales and *Ganoderma* P. Karst is the most speciose genus in the family (Hapuarachchi et al. 2016a, Hapuarachchi et al. 2017, He et al. 2019). Before the molecular era, Polyporales with double-walled basidiospores with a pigmented endosporium ornamented with columns or ridges and a smooth hyaline exosporium were usually placed in Ganodermataceae (Moncalvo and Ryvarden 1997). This family is comprised of ten genera: *Amauroderma* Murrill, *Amaurodermellus* Costa-Rezende, Drechsler-Santos & Góes-Neto, *Foraminispora* Robledo, Costa-Rezende & Drechsler-Santos, *Furtadomyces* Leonardo-Silva, Cotrim & Xavier-Santos, *Ganoderma* P. Karst, *Haddowia* Steyaert, *Humphreya* Steyaert, *Sanguinoderma* Y.F. Sun, D.H. Costa & B.K. Cu,*Tomophagus* Murrill and *Trachyderma* Imazeki (Richter et al. 2014, Costa-Rezende et al. 2017, Costa-Rezende et al. 2020, Sun et al. 2020, Leonardo-Silva et al. 2022). However, Ganodermataceae has been treated as a synonym of Polyporaceae (Justo et al. 2017). There have been several discrepancies regarding the treatment of Justo et al. (2017); in particular, the studied collection of Ganodermatoid specimens was insufficient to establish a stable taxonomic and systematic placement in a phylogenetic context because some herbarium materials have been destroyed or cannot be found, lacking molecular and morphological data and the characterised double-walled basidiospores in Ganodermataceae are quite different from those in Polyporaceae (Cui et al. 2019, Costa-Rezende et al. 2020). In this study, we subsequently followed Justo et al. (2017) since the phylogenetic analyses are more convincing and objective than morphological results.

*Ganoderma* was introduced by Karsten (1881) and typified by *G.lucidum* (Curtis) P. Karst. (syn. *Polyporuslucidus*; bas. *Boletuslucidus* Curtis), a species with stipitate and laccate white-rot Polypore fungi (Karsten 1881, Pegler and Young 1973, Moncalvo and Ryvarden 1997, Keypour et al. 2020). The membership of *Ganoderma* has been subsequently extended, including species with sessile, non-laccate basidiocarps and pigmented, ellipsoid to ovoid, ornamented, double-walled basidiospores (Murrill 1902, Pegler and Young 1973, Steyaert 1980, Cao and Yuan 2013, Papp 2016). Moncalvo and Ryvarden (1997) accepted 148 *Ganoderma* species before the molecular era, of which 65% are recognised as only one or some species, but represented different morphology-based species (Ryvarden 2000, Smith and Sivasithamparam 2003, Torres-Torres and Guzmán-Dávalos 2012). Recently, 180 species of *Ganoderma* were accepted, whereas nearly 500 species are estimated worldwide, of which 60% are awaiting discovery (He et al. 2019, He et al. 2022).

Despite their economic importance, the taxonomy of *Ganoderma* remains uncertain due to a slew of confusion and misconceptions. During the past several decades, many species of *Ganoderma* have been delimited, based on the presence of stipe, laccate or non-laccate, the context of pileus and the microscopic characteristics of basidiospores (Chang and Chen 1984, Seo and Kitamot 1998, Wu et al. 2004, Torres-Torres and Guzmán-Dávalos 2012, Zhou et al. 2016, Tchotet-Tchoumi et al. 2019). In general, it is difficult and subjective to identify *Ganoderma* species solely based on morphological evidence, as their phenotypic traits are sensitive to extrinsic factors, such as illumination, ventilation and humidity (Szedlay et al. 1999, Demoulin 2010, Yang and Feng 2013, Hapuarachchi et al. 2019a). Therefore, morphology-based identification brought *Ganoderma* into a state of taxonomic chaos (Smith and Sivasithamparam 2003, Coetzee et al. 2015, López-Peña et al. 2019, Náplavová et al. 2020). Compared to morphology, molecular methods have turned out to be more effective in resolving intraspecific relationships with *Ganoderma* (Yamashita and Hirose 2016, Fryssouli et al. 2020, Gunnels et al. 2020, Jiang et al. 2021, Shen et al. 2021). Phylogenetic markers, such as IGS, nrSSU, ITS, nrLSU, mtSSU, β-TUB, RPB1, RPB2 and TEF1-α sequences, were independently or conjointly used to infer intraspecific relationships within *Ganoderma* (Cao et al. 2012, Zhou et al. 2015, Xing et al. 2018, Hapuarachchi et al. 2019a, Liu et al. 2019, Ye et al. 2019). In particular, the multilocus phylogeny incorporating sequences from ITS, nrLSU, TEF1-α and RPB2 was applied to give a phylogenetic framework for species delimitation in this genus (Xing et al. 2018, Ye et al. 2019, Tchotet-Tchoumi et al. 2019, Wu et al. 2020, He et al. 2021, Cao et al. 2021). Furthermore, some researchers steered using a combination of morphological, chemotaxonomic and molecular strategies to elevate a steady taxonomy for *Ganoderma* and resolve taxonomic ambiguities (Richter et al. 2014, Welti et al. 2015).

*Ganoderma* has a cosmopolitan distribution and most of the species are known from tropical and sub-tropical regions (He et al. 2019). This fungus grows as saprobes or parasites on deciduous and coniferous trees and some of them are considered as plant pathogens that cause basal stem butt rot and root rot (Pinruan et al. 2010, Ding et al. 2020, Mafia et al. 2020, Mohd et al. 2020). Species of *Ganoderma* play an important role in the nutrient mobilisation process of woody plants. They possess lignocellulose–decomposing enzymes with effective mechanisms for bioenergy production and bioremediation (Coetzee et al. 2015, Kües et al. 2015). In the natural environment, a basidiome has the ability to produce innumerable basidiospores that can be spread by air- or rain-driven and insect vectors (Tuno 1999, Kadowaki et al. 2011, Almaguer et al. 2014, Sadyś et al. 2014). The infection of a plant host by pathogenic *Ganoderma* species starts with the landing of the basidiospore on the wound trunk or root, followed by germination and colonisation (Rees et al. 2009, Rees et al. 2012, Hushiarian et al. 2013, Ayin et al. 2019). Basal stem rot caused by *G.boninense* is the main disease that leads to yield losses and death of oil palm, which account for 50% of substantial economic losses to Southeast Asia’s palm oil industry (Hushiarian et al. 2013, Lee and Chang 2016, Midot et al. 2019). Red roots caused by *G.philippii* are a serious disease of commercial *Acaciamangium* in Malaysia and India (Glen et al. 2014). Since different *Ganoderma* species produce different characteristics and pathogenicity, species identification is difficult, which in turn, leads to significant difficulty in disease control (Wong et al. 2012).

*Ganoderma* was first reported from China by Teng (1934), with four species including *G.lucidum* and one variety. More than 80 species have been introduced so far and several extensive studies have been carried out to investigate *Ganoderma* diversity in China, with new species being introduced (Zhao and Zhang 2000, Wu et al. 2004, Dai et al. 2009, Cao et al. 2012, Hapuarachchi et al. 2015, Hapuarachchi et al. 2018c, Wu et al. 2019, Liu et al. 2019). However, the majority of *Ganoderma* species reported from China have not been subjected to systematic studies (Wang 2012, Hapuarachchi et al. 2016b, Hapuarachchi et al. 2018a, Wang et al. 2019). The objective of the present study is to introduce a novel *Ganoderma* species, from Guizhou Province, southwest China, with descriptions, colour photographs, illustrations and a multigene phylogeny.

## Materials and methods

*Ganoderma* samples were collected from Sandong Township, Sandu Shuizu Autonomous County, Guizhou Province, China, during the rainy season of July 2020. They were dried and preserved as outlined in Hapuarachchi et al. (2019b). The materials used in this study were deposited at Guizhou University (**GACP**) and the Herbarium of Kunming Institute of Botany Academia Sinica (**HKAS**).

### Morphological study

Macro-morphological characteristics were described, based on dried material and the photographs provided here. Colour codes (e.g. 8E8) are from Kornerup and Wanscher (1978). Pileus was sectioned with a razor blade and mounted in 5% potassium hydroxide (KOH) solution. Pileipellis, hyphal systems of pileus, basidia and basidiospores were observed and captured using a compound microscope (Leica DM2500) equipped with a camera. Images were measured with Leica Application Suite X (LAS X). In the description section, the number, length, width and length/width ratio of the measured basidiospores are denoted with symbols *n*, *L*, *W* and *Q*, respectively. The Faces of Fungi number was registered by following Jayasiri et al. (2015).

### DNA Extraction, PCR and Sequencing

Genomic DNA was extracted from dried specimens using an HP Fungal DNA Kit (OMEGA, USA) following the protocol of the manufacturer. PCR amplification was performed in a final volume of 50 µl reaction mixture that contained 25 µl 2x BenchTopTM Taq Master Mix (Biomigas), 19 µl distilled water, 2 µl (10 µM) of each primer and 2 µl template DNA. The large subunit ribosomal RNA (LSU), the internal transcribed spacer (ITS), the translation elongation factor (TEF1-α) and the RNA polymerase II second largest subunit (RPB2) were amplified with primer pairs LROR/LR5 (Vilgalys and Hester 1990), ITS5/ITS4 (White et al. 1990), EF1-983F/EF1-1567R (Rehner and Buckley 2017) and RPB2-5f/RPB2-7cR (Liu et al. 1999). PCR amplification reactions were performed with a T100 Thermal Cycler (T100™, Bio-Rad, USA). The procedures used for amplification of ITS were as follows: initial denaturation at 95°C for 3 min, followed by 35 cycles of denaturation at 95°C for 30 s, annealing at 58°C for 30 s, elongation at 72°C for 1 min and a final extension at 72°C for 5 min. The cycling conditions of LSU, TEF1-α and RPB2 consisted of initial denaturation at 95°C for 3 min, followed by 35 cycles of denaturation at 95°C for 30 s, annealing at 56°C for 30 s, elongation at 72°C for 1.3 min and a final extension at 72°C for 10 min. PCR products were verified by 1% agarose gel electrophoresis and sent to Sangon Biotech (Shanghai, China) for purification and sequencing.

### Sequence Alignment and Phylogenetic Analysis

The raw sequences generated in this study were assembled with ChromasPro (2.1.8). Megablast analysis was conducted using the assembled ITS and RPB2 sequences as the query to check the closely-related taxa. The taxa used in our phylogenetic analysis were selected, based on megablast results and related publications (Table 1). Alignments were performed using MAFFT v. 7 (http://mafft.cbrc.jp/alignment/server/index.html, Katoh and Standley 2013). The resulting alignments were improved manually when necessary, using BioEdit v. 7.0.5.2 (Hall 1999). The introns in TEF and RPB2 were removed, based on the published CDS sequence in GenBank. The aligned ITS1, 5.8S, ITS2, LSU, TEF1-α and RPB2 sequences were concatenated with SequenceMatrix v.1.7.8 (Vaidya et al. 2011). Maximum Likelihood (ML) analysis was performed using RAxMLHPC2 (Stamatakis 2014) on the CIPRES Science Gateway v. 3.3 (Miller et al. 2010). The phylogenetic tree was inferred from four gene-partition analyses, using the GTRCAT model with 25 categories, with settings that the number of bootstrap replicates to 1,000. PartitionFinder v.2 (Lanfear et al. 2017) was used to estimate the best-fit model of nucleotide evolution, with the dataset subdivided into 10 data partitions (TEF 1^st^ codon positions, TEF 2^nd^ codon positions and TEF 3^rd^ codon positions; RPB2 1^st^ codon positions, RPB2 2^nd^ codon positions and RPB2 3^rd^ codon positions; ITS1; 5.8S; ITS2; LSU) and the following settings: branch lengths = unlinked, models = all, model_selection = AICc and search = greedy. Bayesian Inference (BI) analysis was performed in the CIPRES Science Gateway using MrBayes on XSEDE v. 3.2.7a. The GTR+F+I+G4 (TEF 1^st^ codon positions, TEF 2^nd^ codon positions, RPB2 1^st^ codon positions, RPB2 2^nd^ codon positions, LSU and 5.8S), GTR+F+G4 (TEF 3^rd^ codon positions), GTR+F+G4 (RPB2 2^nd^ codon positions), SYM+G4 (ITS1 and ITS2) were selected as the best model. Two runs of four chains were run until the average standard deviation of split frequencies dropped below 0.01, which occurred after 2,360,000 generations. Tree was sampled every 1000th generation and the chain temperature was decreased to 0.05 to improve convergence. The convergence of the runs was checked using TRACER v.1.6 (Rambaut et al. 2013). The first 25% of the resulting samples were discarded as burn-in and posterior probabilities were calculated from the remaining sampled trees (Larget and Simon 1999). In both ML and BY analyses, *Foraminisporaconcentrica* (Cui 12644) and *Foraminisporayinggelingensis* (Cui 13618) were selected as the outgroup (Sun et al. 2020). ML bootstrap values and BY posterior probabilities greater than or equal to 70% and 0.95, respectively, were considered significant support. The phylogenetic tree was visualised with FigTree version 1.4.0 available at http://tree.bio.ed.ac.uk/software/figtree/ (Rambaut 2012).

## Taxon treatments

### Ganoderma ovisporum

H.D. Yang, T.C. Wen sp. nov.

A07C86F9-799E-59BF-AF73-120508E42B3F

IF558589

FoF 10099

#### Materials

- **Type status:** Holotype. **Occurrence:** recordedBy: Hongde Yang; occurrenceID: HKAS123193; **Taxon:** scientificName: *Ganodermaovisporum*; kingdom: Fungi; phylum: Basidiomycota; class: Agaricomycetes; order: Polyporales; family: Polyporaceae; genus: Ganoderma; **Location:** country: China; countryCode: CN; stateProvince: Guizhou; county: Sandu Shuizu Autonomous County; locality: Sandong Township; verbatimElevation: 612 m; verbatimLatitude: 25°70′ N; verbatimLongitude: 107°96′ E; **Identification:** identifiedBy: Hongde Yang; **Event:** year: 2020; month: July; day: 16; habitat: Terrestrial; fieldNotes: Rotten wood, in dry dipterocarp forest and in upper mixed deciduous forest and growing up from soil; **Record Level:** type: HKAS123193; collectionID: SD2020071601

#### Description

**Figure 1.**
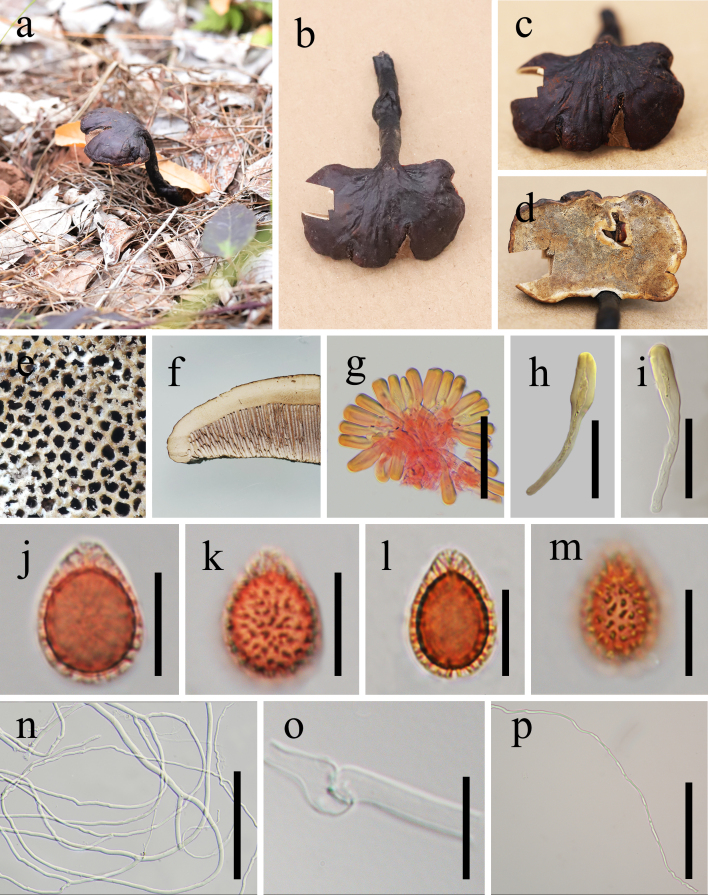
*Ganodermaovisporum* (HKAS123193, holotype). **a**–**b** Basidiome; **c** Pileus; **d** Pore surface; **e** Pores; **f** Sections of pileus; **g**–**i** Pileipellis terminal cell; **j–m** Basidiospores; **n** Skeletal hyphae; **o** Generative hyphae; **p** Binding hyphae. Scale bars: g = 50 µm; h–i = 30 µm; j–m = 10 µm; n = 100 µm; o = 10 µm; p = 100 µm.

Basidiome annual, stipitate, corky, strongly laccate, becoming lighter when dry. Pileus 3 × 5 cm, up to 0.9 cm thick at the base, applanate, subreniform, upper surface red-brown (8E8) when fresh, becoming brownish-black (6C8) when dry, with slightly concentrically sulcate, radially rugose, irregularly tuberculate bumps and ridges overlying the context. Margin is slightly obtuse, yellow-brown (5D8) or concolorous with the pileus. Pore surface pale white (4A2). Pores nearly round to round, 3–4 per mm, dissepiments thin to slightly thick. Context up to 0.3 cm thick, corky, homogeneous at the periphery, becoming three-layered towards the centre, upper layer creamy-white (6E4), middle layer pale brown (6E4), lower layer brown (6D1), without concentric growth zone, black melanoid band absent. There is a line of independent or confluent, laterally arranged tubes inserted between the upper and middle layers of the context. Tubes up to 0.6 cm long, brownish (6E7). Stipe slightly darker than pileus, lateral, subcylindrical, 4-7 cm long, up to 1 cm in diam. Basidia not observed. Basidiospores (12.5–)13.0–13.5–15.0(–15.5) × (9.0–)9.5–10.0–10.5(–11.5) μm (Q_m_ = 1.3, Q = 1.0–1.7，n = 30, with myxosporium), ellipsoid to broadly ellipsoid, ovoid, brown, double-walled, with a dark brown eusporium bearing coarse echinulae and an obtuse turgid appendix, overlaid by a hyaline, smooth myxosporium. Pileipellis hymeniodermiformic, yellowish-brown, terminal cells clavate, entire, brown (5D6), thick-walled, hollow, 18–29 × 6–11 μm. Hyphal system trimitic, generative hyphae 3.5–6 μm in diam., hyaline, colourless, thin-walled with clamp connections; skeletal hyphae 3–6 μm in diam., thick-walled to nearly solid, sometimes branched; binding hyphae 1.5–3 μm in diam., thick-walled, nearly solid, colourless (Fig. 1).

#### Etymology

Referring to the ovoid basidiospores.

#### Notes

*Ganodermaovisporum* clusters with *G.sandunense* in the multigene phylogenetic tree (Fig. 3), the former is similar to the latter by having 98% and 97% homology in ITS and RPB2 sequence data, respectively. These two species are similar in having wide ovoid basidiospores and inhabiting deciduous coniferous mixed forests. However, *G.ovisporum* differs from *G.sandunense* in having inconspicuously concentric rings near the pileus margin, lateral stipe and shorter pileipellis terminal cells (18–29 × 6–11 μm), while conspicuously concentric zones and vertically-arranged ridges or grooves, central stipe and longer pileipellis terminal cells (50–95 × 8–13.5 μm) have been observed in the latter. By considering both phylogenetic evidence and morphological observations, we conclude our collection is a new species in *Ganoderma*.

### Ganoderma sandunense

Hapuar., T.C. Wen & K.D. Hyde

EA035F7B-6ED1-584B-80C8-CC61BBA7305C

IF555784

FoF05659

#### Materials

- **Type status:** Holotype. **Occurrence:** recordedBy: Ting-Chi Wen; occurrenceID: GACP18012501; **Taxon:** scientificName: *Ganodermasandunense*; kingdom: Fungi; phylum: Basidiomycota; class: Agaricomycetes; order: Polyporales; family: Polyporaceae; genus: Ganoderma; **Location:** country: China; countryCode: CN; stateProvince: Guizhou; county: Sandu Shuizu Autonomous County; verbatimElevation: 590 m; verbatimLatitude: 24°54′N; verbatimLongitude: 107°53′E; **Identification:** identifiedBy: Kalani Hapuarachchi; **Event:** year: 2018; month: January; day: 25; habitat: Terrestrial; fieldNotes: Rotten wood, growing up from the soil; **Record Level:** type: GACP18012501; collectionID: GACP18012501

#### Description

**Figure 2.**
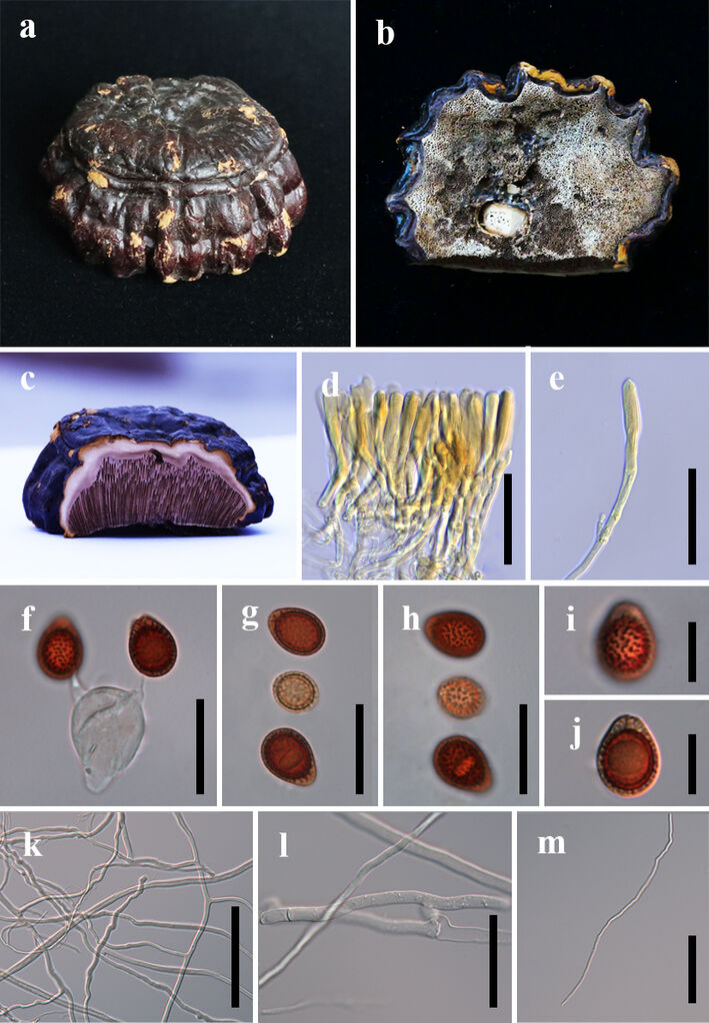
*Ganodermasandunense* (GACP18012501, holotype). **a** Basidiome; **b** Pore surface; **c** Sections of pileus; **d**–**e** Pileipellis terminal cell; **f** Basidia; **g**–**j** Basidiospores; **k** Skeletal hyphae and binding hyphae; **l** Generative hyphae; **m** Binding hyphae. Scale bars: d–e= 50 μm; f–h = 20 μm; i–j = 10 μm; k = 100 μm; l–m = 50 μm.

Basidiome annual, stipitate, corky, strongly laccate. Pileus hemispherical, projecting 8 cm, up to 4 cm wide and 1.5 cm thick. Pileal surface reddish-black (8E8) to brownish-black (6C8), with distinctly concentrically sulcate, vertically-arranged ridges or grooves. Margin obtuse, concolorous with the pileus. Pore surface whitish-yellow (4A2) to light brown (6D4). Pores nearly circular, 3–5 per mm, dissepiments thin. Context up to 0.5 cm thick, inconspicuous triplex, fawn (5C5) to creamy-white (5A1) to dark brown (5E6), without concentric growth zone, black melanoid band absent. There is a line of independent or confluent, laterally-arranged tubes inserted between the upper and middle layer of the context. Tubes up to 1.2 cm long, dark brown (7F8). Stipe slightly darker than pileus, central, subcylindrical, up to 8 cm, 0.5 cm in diam. Basidia broadly ellipsoid, 21–25.5 × 13.5–17.5 μm, with four sterigmata. Basidiospores (12.3–)13.2–13.7–14.2(–15.7) × (9.0–)10–10.3–10.6(–12.5) μm (Qm = 1.3, Q = 1.0–1.7, n = 30, with myxosporium), ellipsoid to broadly ellipsoid, brown (7E5). Pileipellis cells clavate like, entire, brownish-orange (5C5), 50–95 × 8–13.5 μm. Hyphal system trimitic, generative hyphae 4-6 μm in diam., hyaline, colourless, thin-walled with clamp connections; skeletal hyphae 3.5–6 μm in diam., thick-walled to nearly solid, sometimes branched; binding hyphae 1-2 μm in diam., thick-walled, nearly solid, colourless (Fig. 2).

#### Notes

*Ganodermasandunense* was introduced by Hapuarachchi et al. (2019b) with ITS sequence. In addition, the description of its basidia is absent in their publication. In this study, the holotype of *G.sandunense* was loaned from Herbarium (**GACP**) and re-examined. We have refined this species with a more detailed illustration. Furthermore, we provided RPB2 sequence data of this species, which is an important phylogenetic marker used for intraspecific delimitation within *Ganoderma*.

## Identification Keys

### Keys to 22 species of laccate *Ganoderma* species in China

**Table:** 

1	Distributed in China with gymnosperms as substrates	*G.tsugae*
–	Distributed in China with angiosperms as substrates	2
2	Basidiome sessile	3
–	Basidiome stipitate to substipitate	5
3	Pileipellis terminal cells regular, clavate, occasionally with blunt outgrowth and protuberance, context present melanoid bands, basidiospores 8–12 × 3.8–5.2 µm	*G.angustisporum*
–	Pileipellis terminal cells are irregular, mainly composed of clavate cells or branched cells with blunt outgrowths in the lateral part or protuberances in the apical	4
4	Melanoid bands absent in the context, concentric growth zones present in the context, basidiospores 9.2–12 × 6.8–8.4 μm	*G.mutabile*
–	Melanoid bands present in the context, concentric growth zones absent in the context, basidiospores 8–13.5 × 4.2–6.3 μm	*G.boninense*
5	Distributed in tropical regions	6
–	Distributed mainly in temperate regions	8
6	Basidiome notably with a long, lateral stipe, pileus smaller, basidiospores with coarsely echinulate, 8.5–11 × 5–7 μm	*G.flexipes*
–	Basidiome stipitate to substipitate, pileus dimidiate, mostly large	7
7	Pileus single or occasionally composed of many small pilei, concentric growth zones present in the context, basidiospores with fine and long echinulate, 8–11.3 × 5–12.8 μm	*G.multipileum*
–	Pileus is mostly single, concentric growth zones absent in the context, basidiospores with coarse and short echinulae, 8.5–12.5 × 5.5–7.5 μm	*G.orbiforme*
8	Context nearly homogeneous to homogeneous	9
–	Context duplex to triplex	12
9	Pileus context white, pore surface white to cream, basidiospores 9.5–12.5 × 7–9 μm	*G.leucocontextum*
–	Pileus context brownish to brown or darker	10
10	Pileipellis terminal cells are mostly irregular, context present melanoid bands and concentric growth zones, basidiospores 10.8–13.1 × 8.3–11 μm	*G.tropicum*
–	Pileipellis terminal cells regular, cylindrical to clavate, context absent melanoid bands	11
11	Inhabiting deciduous forests, basidiospores ellipsoid, normally with an orderly arranged echinulae, basidiospores 10.7–12.8 × 7.0–9.0 μm	*G.sinense*
–	Inhabiting bamboo forests	12
12	Pileipellis terminal cells 35–65 × 8–16 μm, basidiospores 11–12.5 × 6.5–7.5 μm	*G.bambusicola*
–	Pileipellis terminal cells 20–55 × 10–15 μm, basidiospores 8.0–12.5 × 5.0–8.0 μm	*G.esculentum*
13	Chlamydospores present in the context, basidiospores 7.8–10.4 × 5.2–6.4 μm	*G.weberianum*
–	Chlamydospores absent in context	14
14	Basidiospores < 8 μm in width and < 12 μm in length	15
–	Basidiospores > 8 μm in width and > 9 μm in length	17
15	Basidiome corky, context soft, pores 2–4 per mm, pileipellis terminal cells regular, clavate, 20‒35 × 10‒12 μm, basidiospores 5.7‒8.3 × 2.6‒4.6 μm	*G.weixiensis*
–	Basidiome corky to woody, context firm, pores 4–6 per mm, pileipellis terminal cells occasional with outgrowths	16
16	Growing on living trees of *Casuarinaequisetifolia*, pileipellis terminal cells 40–70 × 5–13 µm, basidiospores 8.3–11.5 × 4.5–7 µm	*G.casuarinicola*
–	Growing on deciduous trees, pileipellis terminal cells 20–40 × 7–15 μm, basidiospores 7–9.3 × 4.6–6.8 μm	*G.lingzhi*
17	Basidiospores ellipsoid, with sinuous ridge-like echinulae, 12.3–13.8 × 8.5–9.8 μm	*G.lucidum*
–	Basidiospores broadly ellipsoid, with coarse echinulae and an obtuse turgid appendix	18
18	Context brown to dark brown	19
–	Context greyish-white to fawn brown	20
19	Pores 4–5 per mm, pileipellis terminal cells 25–30 × 7.5–8.5 μm, basidiospores 11.0–13.0 × 8.0–9.5 μm	*G.shanxiense*
–	Pores 5–8 per mm, pileipellis terminal cells 20–45 × 5.5–7.5 μm, basidiospores 9.0–12.5 × 6.5–9.0 μm	*G.dianzhongense*
20	Distributed in Shandong Province, pileipellis terminal cells 17–25 × 4.5–7.5 μm, basidiospores 9‒13 × 6‒9 μm	*G.shandongense*
–	Distributed in Guizhou Province	21
21	Basidiome with a central stipe, pileipellis terminal cells 50–95 × 8–13.5 μm, basidiospores 12.3–15.7 × 9.1–12.0 μm	*G.sandunense*
–	Basidiome with a lateral stipe, pileipellis terminal cells 18–29 × 6–11 μm, basidiospores 12.5–15.5 × 9.0–11.5 μm	*G.ovisporum*

## Analysis

### Phylogenetic analyses

**Figure 3.**
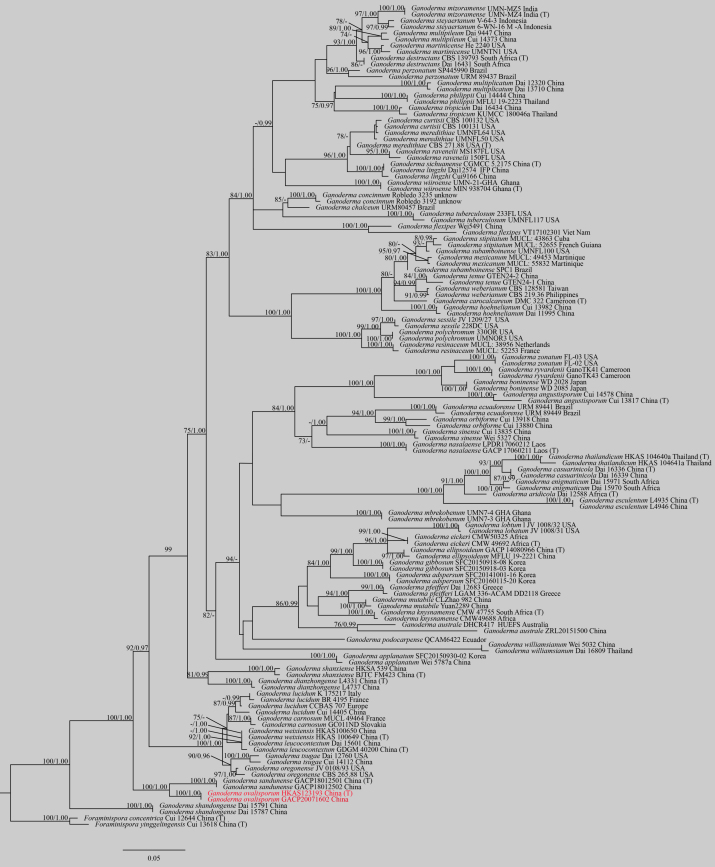
Phylogram for *Ganoderma* generated from Maximum Likelihood analysis of ITS, LSU, TEF1-α and RPB2 sequence data. Bootstrap support values for Maximum Likelihood and maximum parsimony greater than 70% and posterior probabilities of Bayesian Inference ≥ 0.95 are given above branches. Type specimens are marked with letter (T) and new species in this study are indicated in red.

Eight sequences of ITS, LSU and RPB2 were successfully amplified, but we failed to obtain the TEF1-α sequence from the two specimens HKAS123193 and GACP20071602. The newly-generated sequences and sequences from GenBank represented 132 specimens from 66 species, of which 21 were the type. The combined alignment of sequences comprised 3028 characters of 606, 1020, 809, 593 belonging to TEF1-α, RPB2, ITS and LSU, respectively. The final ML optimisation log-likelihood was -17354.28. The Bayesian Inference stopped at 2915000 generations when the average standard deviation of split frequencies reached 0.009904. The tree topologies derived from ML and BY were identical. Therefore, only the ML tree is shown (Fig. 3). The new species *G.ovisporum* and *G.sandunense* formed an individual clade in the phylogenetic tree (Fig. 3).

## Discussion

In this study, both phylogeny and morphology support *G.ovisporum* as a new species. Morphologically, it resembles other dark-coloured, laccate, stipitate *Ganoderma* species. However, it can be distinguished by having larger (12.5–15.5 × 9.0–11.5 μm), wide ovoid, dark brown-pigmented basidiospores. It is mostly similar to *G.sandunense* in having brownish-black pileus and similarly-sized basidiospores, as well distribution in Guizhou Province (Hapuarachchi et al. 2019b). The former species is distinct from the latter by having a lateral stipe and shorter pileipellis terminal cells (18–29 × 6–11 μm). Phylogenetically, *G.ovisporum* and *G.sandunense* are closely related, forming a distinct clade with basal position with strong support.

*Ganoderma* was extensively researched by the Chinese because it applied to medicine and food, together with the symbolic happiness and immortality culture, those being recognised as long as 2,000 years ago (Hapuarachchi et al. 2016b, Hapuarachchi et al. 2018b, Li et al. 2018, Cui et al. 2019, Du et al. 2021). Chinese taxonomists emphasised the morphological characteristics, such as stipe, pileus, pores, context, pileipellis terminal cells and basidiospores as keys to identity (Zhao 1989, He and Yu 1989, Zhang 1997). Keeping this method, Zhao and Zhang (2000) recorded 76 *Ganoderma* species from China, providing detailed illustrations. Wu and Dai (2005) identified 77 *Ganoderma* species with full description and colour photographs. Studies have been implemented to revise the taxonomy of *Ganoderma* in China by using molecular and morphology methods in the recent decade. The results indicated at least 23 species names are synonyms and confirmed that 24 species are distributed in China, 16 of which possess laccate basidiomes (Wang 2012, Chao 2013, Xing 2019). Since then, six species with laccate basidiomes have been described from China: *G.bambusicola*, *G.dianzhongense*, *G.esculentum*, *G.sandunense*, *G.shanxiense* and *G.weixinense* (Hapuarachchi et al. 2019b, Liu et al. 2019, Ye et al. 2019, Wu et al. 2020, He et al. 2021). *Ganoderma* taxonomy has undergone tremendous changes since both phenotypic features and phylogeny were used to delineate species (Gottlieb et al. 2000, Hapuarachchi et al. 2018c, Hapuarachchi et al. 2018a, Lin and Yang 2019, Tchotet-Tchoumi et al. 2019, He et al. 2022). Based on the aforementioned characteristics, we have provided a dichotomous key to 22 laccate species, including our new species from China.

*Ganoderma* could originate from Southeast Asia and later dispersal to the Northern Hemispheres, the Southern Hemispheres and the neotropics before 30 Mya years, during which species radiation and diversification events happened (Moncalvo and Buchanan 2008). Overviewing *Ganoderma* species worldwide, Imazeki (1939) concluded using subgenera *Euganoderma* and *Elfvingia* to accommodate species with laccate and non-laccate characters, respectively. In this study, a phylogenetic analysis was carried out using combined LSU, ITS, TEF1-α and RPB2 sequences from 66 species that included species previously placed in the above two subgenera. The topology of our phylogenetic tree is consistent with the morphology that the laccate species and non-laccate species tend to form groups. It is worth mentioning that the new species *G.ovisporum* group with the laccate species of *G.carnosum*, *G.dianzhongense*, *G.leucocontextum*, *G.lucidum*, *G.oregonense*, *G.sandunense*, *G.shandongense*, *G.shanxiense*, *G.tsugae* and *G.weixiensis* had strong support in both ML and Bayesian analyses. Those species were found in only or few ecological niches, except the widely cultivated *G.leucocontextum*, *G.lucidum and G.tsugae* (Gottlieb et al. 2000, Moncalvo and Buchanan 2008, Hapuarachchi et al. 2018b, Lin and Yang 2019). Therefore, many *Ganoderma* species are geographically restricted (He et al. 2022). However, the phylogenetic tree in the case of the laccate species *G.pfeifferi* and *G.mutabile* grouped with the non-laccate species *G.adspersum*, *G.australe*, *G.eickeri*, *G.ellipsoideum*, *G.gibbosum*, *G.knysnamense*, *G.lobatum*, *G.podocarpense* and *G.williamsianum*, indicating *Euganoderma* and *Elfvingia* are polyphyletic (Gottlieb et al. 2000). However, in fact, they are similar in having a substipitate to sessile basidiome and living as saprobes or parasites (Hapuarachchi et al. 2018c, Tchotet-Tchoumi et al. 2019). Consequently, biogeographic patterns and convergent evolution could explain the population structure and evolution of *Ganoderma*. Thus, a phylogeography study would help better understand the evolution of *Ganoderma*.

## Supplementary Material

XML Treatment for Ganoderma ovisporum

XML Treatment for Ganoderma sandunense

## Figures and Tables

**Table 1.** The species, specimens and GenBank accession numbers of sequences used in this study

Species	Voucher	Geographic origin	GenBank accession numbers				References
			ITS	LSU	EF-1	RPB2	
*G.adspersum*	SFC20141001-16	Korea	KY364251	–	KY393284	KY393270	Jargalmaa et al. (2017)
*G.adspersum*	SFC20160115-20	Korea	KY364254	–	KY393286	KY393272	Jargalmaa et al. (2017)
*G.angustisporum*	Cui 14578	China	MG279171	–	MG367564	–	Xing et al. (2018)
*G.angustisporum*	Cui 13817 (T)	China	MG279170	–	MG367563	MG367507	Xing et al. (2018))
*G.applanatum*	SFC20150930-02	Korea	KY364258	–	KY393288	KY393274	Jargalmaa et al. (2017)
*G.applanatum*	Wei 5787a	China	KF495001	–	KF494978	–	GenBank
*G.aridicola*	Dai 12588 (T)	Africa	KU572491	–	KU572502	–	Xing et al. (2016)
*G.australe*	DHCR417 (HUEFS)	Australia	MF436676	MF436673	MF436678	–	Costa-Rezende et al. (2017)
*G.australe*	ZRL20151500	China	LT716076	KY418900	KY419088	–	Zhao et al. (2017)
*G.boninense*	WD 2085	Japan	KJ143906	–	KJ143925	KJ143965	Zhou et al. (2015)
*G.boninense*	WD 2028	Japan	KJ143905	KU220015	KJ143924	–	Zhou et al. (2015)
*G.carnosum*	MUCL 49464	France	MG706220	MG706168	MG837838	MG837793	GenBank
*G.carnosum*	GC011ND	Slovakia	MK415266	MK995647		–	Náplavová et al. (2020)
*G.carocalcareum*	DMC 322 (T)	Cameroon	EU089969	–	–	–	Douanla-Meli and Langer (2009)
*G.casuarinicola*	Dai 16339	China	MG279176	–	MG367568	MG367511	Xing et al. (2018)
*G.casuarinicola*	Dai 16336 (T)	China	MG279173	–	MG367565	MG367508	Xing et al. (2018)
*G.chalceum*	URM80457	Brazil	JX310812	JX310826	–	–	GenBank
*G.concinnum*	Robledo 3235	–	MN077523	MN077557	–	–	Costa-Rezende et al. (2020)
*G.concinnum*	Robledo 3192	–	MN077522	MN077556	–	–	Costa-Rezende et al. (2020)
*G.curtisii*	CBS 100131	USA	JQ781848	–	KJ143926	KJ143966	Zhou et al. (2015)
*G.curtisii*	CBS 100132	USA	JQ520164	–	KJ143927	KJ143967	Zhou et al. (2015)
*G.destructans*	CBS 139793 (T)	South Africa	NR_132919	NG_058157	–	–	Coetzee et al. (2015)
*G.destructans*	Dai 16431	South Africa	MG279177	–	MG367569	MG367512	Xing et al. (2018)
*G.dianzhongense*	L4331(T)	China	MW750237	–	–	MZ467043	He et al. (2021)
*G.dianzhongense*	L4737	China	MW750238	–	–	MW839000	He et al. (2021)
*G.ecuadorense*	URM 89449	Brazil	MK119828	MK119908	MK121577	MK121535	Sun et al. (2020)
*G.ecuadorense*	URM 89441	Brazil	MK119827	MK119907	MK121576	MK121534	Sun et al. (2020)
*G.eickeri*	CMW50325	Africa	MH571689	–	MH567290	–	Tchotet-Tchoumi et al. (2019)
*G.eickeri*	CMW 49692 (T)	Africa	NR_165524	–	–	–	Tchotet-Tchoumi et al. (2019)
*G.ellipsoideum*	MFLU 19-2221	China	MN398339	MN428664	MN423157	–	GenBank
*G.ellipsoideum*	GACP 14080966 (T)	China	NR_160617	–	–	–	Hapuarachchi et al. (2018c)
*G.enigmaticum*	Dai 15971	South Africa	KU572487	–	KU572497	MG367514	Xing et al. (2016)
*G.enigmaticum*	Dai 15970	South Africa	KU572486	–	KU572496	MG367513	Xing et al. (2016)
*G.esculentum*	L4935 (T)	China	MW750242	–	–	MW839004	He et al. (2021)
*G.esculentum*	L4946	China	MW750243	–	–	–	He et al. (2021)
*G.flexipes*	VT17102301	Viet Nam	MK345430	MK346830	–	–	Hapuarachchi et al. (2019b)
*G.flexipes*	Wei5491	China	JQ781850	–	–	KJ143968	Cao et al. (2012)
*G.gibbosum*	SFC20150918-08	Korea	KY364271	–	KY393291	KY393278	Jargalmaa et al. (2017)
*G.gibbosum*	SFC20150918-03	Korea	KY364270	–	KY393290	KY393277	Jargalmaa et al. (2017)
*G.hoehnelianum*	Dai 11995	China	KU219988	KU220016	MG367550	MG367497	Xing et al. (2018)
*G.hoehnelianum*	Cui 13982	China	MG279178	–	MG367570	MG367515	Xing et al. (2018)
*G.knysnamense*	CMW 47755 (T)	South Africa	NR_165523	–	MH567261	–	Tchotet-Tchoumi et al. (2019)
*G.knysnamense*	CMW49688	Africa	MH571683	–	MH567266	–	Tchotet-Tchoumi et al. (2019)
*G.leucocontextum*	Dai 15601	China	KU572485	–	KU572495	MG367516	Xing et al. (2016)
*G.leucocontextum*	GDGM 40200 (T)	China	KM396272	–	–	–	Li et al. (2015)
*G.lingzhi*	Dai12574 (IFP)	China	KJ143908	–	JX029977	JX029981	Zhou et al. (2015)
*G.lingzhi*	Cui9166	China	KJ143907	–	JX029974	JX029978	Cao et al. (2012)
*G.lobatum*	JV 1008/31	USA	KF605671	–	MG367553	MG367499	Xing et al. (2018)
*G.lobatum*	JV 1008/32	USA	KF605670	–	MG367554	MG367500	Xing et al. (2018)
*G.lucidum*	BR 4195	France	KJ143909	–	–	KJ143969	Zhou et al. (2015)
*G.lucidum*	K 175217	Italy	KJ143911	–	KJ143929	KJ143971	Zhou et al. (2015)
*G.lucidum*	Cui 14405	China	MG279182	–	MG367574	MG367520	Xing et al. (2018)
*G.lucidum*	CCBAS 707	Europe	MG706231	MG706177	MG837846	MG837805	GenBank
*G.martinicense*	UMNTN1	USA	MG654178	–	MG754738	MG754860	Loyd et al. (2018)
*G.martinicense*	He 2240	USA	MG279163	–	MG367557	MG367503	Xing et al. (2018)
*G.mbrekobenum*	UMN7-4 GHA	Ghana	KX000898	KX000899	–	–	Crous et al. (2016)
*G.mbrekobenum*	UMN7-3 GHA	Ghana	KX000896	KX000897	–	–	Crous et al. (2016)
*G.meredithiae*	UMNFL50	USA	MG654103	–	MG754735	MG754862	Loyd et al. (2018)
*G.meredithiae*	CBS 271.88 (T)	USA	NR_164435	NG_067432	–	–	Vu et al. (2019)
*G.meredithiae*	UMNFL64	USA	MG654188	–	MG754734	MG754861	Loyd et al. (2018)
*G.mexicanum*	MUCL: 55832	Martinique	MK531815	–	MK531829	MK531839	Cabarroi-Hernández et al. (2019)
*G.mexicanum*	MUCL: 49453	Martinique	MK531811	–	MK531825	MK531836	Cabarroi-Hernández et al. (2019)
*G.mizoramense*	UMN-MZ5	India	KY643751	–	–	–	Crous et al. (2017)
*G.mizoramense*	UMN-MZ4 (T)	India	KY643750	–	–	–	Crous et al. (2017)
*G.multipileum*	Cui 14373	China	MG279184	–	MG367575	MG367521	Xing et al. (2018)
*G.multipileum*	Dai 9447	China	KJ143914	–	KJ143932	KJ143973	Zhou et al. (2015)
*G.multiplicatum*	Dai 12320	China	KU572490	–	KU572500	–	Xing et al. (2016)
*G.multiplicatum*	Dai 13710	China	KU572489	–	KU572499	–	Xing et al. (2016)
*G.mutabile*	Yuan2289	China	JN383977	–	–	–	Cao and Yuan (2013)
*G.mutabile*	CLZhao 982	China	MG231527	–	–	–	Cao and Yuan (2013)
*G.nasalaense*	LPDR17060212	Laos	MK345442	MK346832	–	–	Hapuarachchi et al. (2019b)
*G.nasalaense*	GACP 17060211 (T)	Laos	NR_164048	NG_066439	–	–	Hapuarachchi et al. (2019b)
*G.orbiforme*	Cui 13880	China	MG279187	–	MG367577	MG367523	Xing et al. (2018)
*G.orbiforme*	Cui 13918	China	MG279186	–	MG367576	MG367522	Xing et al. (2018)
*G.oregonense*	JV 0108/93	USA	KF605620	–	MG367558	MG367504	Xing et al. (2018)
*G.oregonense*	CBS 265.88	USA	JQ781875	–	KJ143933	KJ143974	Zhou et al. (2015)
*G.ovisporum*	HKAS123193 (T)	China	**MZ519547**	**MZ519545**	–	**MZ547661**	This study
*G.ovisporum*	GACP20071602	China	**MZ519548**	**MZ519546**	–	**MZ547662**	This study
*G.perzonatum*	URM 89437	Brazil	MK119830	–	MK121579	–	Sun et al. (2020)
*G.perzonatum*	SP445990	Brazil	KJ792750	–	–	–	GenBank
*G.pfeifferi*	LGAM 336-ACAM DD2118	Greece	MG706232	MG706178	MG837847	MG837806	GenBank
*G.pfeifferi*	Dai 12683	Greece	MG279165	–	MG367560	–	Xing et al. (2018)
*G.philippii*	Cui 14444	China	MG279189	–	MG367579	MG367525	Xing et al. (2018)
*G.philippii*	MFLU 19-2223	Thailand	MN401411	MN398327	MN423175	–	GenBank
*G.podocarpense*	QCAM6422	Ecuador	MF796661	MF796660	–	–	GenBank
*G.polychromum*	330OR	USA	MG654196	–	MG754742	–	Loyd et al. (2018)
*G.polychromum*	UMNOR3	USA	MG654204	–	MG754744	–	Loyd et al. (2018)
*G.ravenelii*	MS187FL	USA	MG654211	–	MG754745	MG754865	Loyd et al. (2018)
*G.ravenelii*	150FL	USA	MG654207	–	–	–	Loyd et al. (2018)
*G.resinaceum*	MUCL: 38956	Netherlands	MK554772	–	MK554723	MK554747	Cabarroi-Hernández et al. (2019)
*G.resinaceum*	MUCL: 52253	France	MK554786	–	MK554737	MK554764	Cabarroi-Hernández et al. (2019)
*G.ryvardenii*	GanoTK41	Cameroon	JN105699	–	–	–	Kinge et al. (2012)
*G.ryvardenii*	GanoTK43	Cameroon	JN105695	–	–	–	Kinge et al. (2012)
*G.sandunense*	GACP18012502	China	MK345451	–	–	**MZ547664**	Hapuarachchi et al. (2019b)
*G.sandunense*	GACP18012501 (T)	China	NR_164049	–	–	**MZ547663**	Hapuarachchi et al. (2019b)
*G.sessile*	228DC	USA	MG654319	–	MG754750	MG754869	Loyd et al. (2018)
*G.sessile*	JV 1209/27	USA	KF605630	–	KJ143937	KJ143976	Zhou et al. (2015)
*G.shandongense*	Dai 15791	China	MG279192	–	MG367582	MG367528	Xing et al. (2018)
*G.shandongense*	Dai 15787	China	MG279191	–	MG367581	MG367527	Xing et al. (2018)
*G.shanxiense*	HSA 539	China	MK764269	–	–	MK789681	Liu et al. (2019)
*G.shanxiense*	BJTC FM423 (T)	China	MK764268	–	MK783937	MK783940	Liu et al. (2019)
*G.sichuanense*	CGMCC 5.2175 (T)	China	NR_152892	–	–	KC662404	Yao et al. (2013)
*G.sinense*	Cui 13835	China	MG279193	–	MG367583	MG367530	Xing et al. (2018)
*G.sinense*	Wei 5327	China	KF494998	KF495008	KF494976	MG367529	Xing et al. (2018)
*G.steyaertanum*	6-WN-16(M)-A	Indonesia	KJ654461	–	–	–	Glen et al. (2014)
*G.steyaertanum*	V-64-3	Indonesia	KJ654433	–	–	–	Glen et al. (2014)
*G.stipitatum*	MUCL: 52655	French Guiana	MK554770	–	MK554717	MK554755	Cabarroi-Hernández et al. (2019)
*G.stipitatum*	MUCL: 43863	Cuba	MK554769	–	MK554739	MK554745	Cabarroi-Hernández et al. (2019)
*G.subamboinense*	UMNFL100	USA	MG654373	–	MG754762	–	Loyd et al. (2018)
*G.subamboinense*	SPC1	Brazil	KU569546	KU570945	–	–	Bolaños et al. (2016))
*G.tenue*	GTEN24-1	China	DQ424977	–	–	–	GenBank
*G.tenue*	GTEN24-2	China	DQ424978	–	–	–	GenBank
*G.thailandicum*	HKAS 104641a	Thailand	MK848682	MK849880	MK875830	MK875832	Luangharn et al. (2019a)
*G.thailandicum*	HKAS 104640a (T)	Thailand	MK848681	MK849879	MK875829	MK875831	Luangharn et al. (2019a)
*G.tropicum*	Dai 16434	China	MG279194	–	MG367585	MG367532	Xing et al. (2018)
*G.tropicum*	KUMCC 18–0046a	Thailand	MH823539	–	–	MH883621	Luangharn et al. (2019b)
*G.tsugae*	Cui 14112	China	MG279196	–	MG367587	MG367534	Xing et al. (2018)
*G.tsugae*	Dai 12760	USA	KJ143920	–	KJ143940	KJ143978	Zhou et al. (2015)
*G.tuberculosum*	UMNFL117	USA	MG654359	–	MG754771	–	Loyd et al. (2018)
*G.tuberculosum*	233FL	USA	MG654367	–	–	MG754873	Loyd et al. (2018)
*G.weberianum*	CBS 219.36	Philippines	MH855780	MH867289	MK611974	MK611972	Cabarroi-Hernández et al. (2019)
*G.weberianum*	CBS 128581	Taiwan	MH864975	MH876427	MK636693	MK611971	Cabarroi-Hernández et al. (2019)
*G.weixiensis*	HKAS 100649 (T)	China	NR_166271	NG_067863	MK302442	–	Ye et al. (2019)
*G.weixiensis*	HKAS100650	China	MK302445	MK302447	MK302443	–	Ye et al. (2019)
*G.wiiroense*	UMN-21-GHA	Ghana	KT952363	KT952364	–	–	Crous et al. (2015))
*G.wiiroense*	MIN 938704 (T)	Ghana	NR_158480	NG_064392	–	–	Crous et al. (2015)
*G.williamsianum*	Dai 16809	Thailand	MG279183	–	MG367588	MG367535	Xing et al. (2018)
*G.williamsianum*	Wei 5032	China	KU219994	KU220024	–	–	Song et al. (2016)
*G.zonatum*	FL-03	USA	KJ143922	–	KJ143942	KJ143980	Zhou et al. (2015)
*G.zonatum*	FL-02	USA	KJ143921	–	KJ143941	KJ143979	Zhou et al. (2015)
*Foraminisporaconcentrica*	Cui 12644 (T)	China	NR_158325	NG_064396	MK121561	MK121499	Sun et al. (2020)
*F.yinggelingensis*	Cui 13618 (T)	China	NR_174805	MK119900	MK121570	MK121536	Sun et al. (2020)

## References

[B7614662] Almaguer M., Rojas-Flores T. I., Rodríguez-Rajo F. J, Aira M. J (2014). Airborne basidiospores of *Coprinus* and *Ganoderma* in a Caribbean region. Aerobiologia.

[B7614672] Ayin C. M., Alvarez A. M., Awana C., Schleinzer F. M., Marx B. C., Schlub R. L. (2019). *Ralstoniasolanacearum*, *Ganodermaaustrale*, and bacterial wetwood as predictors of ironwood tree (*Casuarinaequisetifolia*) decline in Guam. Australasian Plant Pathology.

[B7614703] Bolaños Ana. Cristina, Bononi Vera. Lúcia. Ramos, Gugliotta Adriana. De. Mello (2016). New records of *Ganodermamultiplicatum* (Mont.) Pat. (Polyporales, Basidiomycota) from Colombia and its geographic distribution in South America. Check List.

[B7614712] Cabarroi-Hernández Milay, Villalobos-Arámbula Alma. Rosa, Torres-Torres Mabel. Gisela, Decock Cony, Guzmán-Dávalos Laura (2019). The *Ganodermaweberianum-resinaceum* lineage: multilocus phylogenetic analysis and morphology confirm *G.mexicanum* and *G.parvulum* in the Neotropics. MycoKeys.

[B7838337] Cao Bin, Haelewaters Danny, Schoutteten Nathan, Begerow Dominik, Boekhout Teun, Giachini Admir J., Gorjón Sergio P., Gunde-Cimerman Nina, Hyde Kevin D., Kemler Martin, Li Guo Jie, Liu Dong Mei, Liu Xin Zhan, Nuytinck Jorinde, Papp Viktor, Savchenko Anton, Savchenko Kyryll, Tedersoo Leho, Theelen Bart, Thines Marco, Tomšovský Michal, Toome-Heller Merje, Urón Judith P., Verbeken Annemieke, Vizzini Alfredo, Yurkov Andrey M., Zamora Juan Carlos, Zhao Rui Lin (2021). Delimiting species in Basidiomycota: a review. Fungal Diversity.

[B7614722] Cao Yun, Wu Sheng. Hua, Dai Yu. Cheng (2012). Species clarification of the prize medicinal *Ganoderma* mushroom “Lingzhi”. Fungal Diversity.

[B7614740] Cao Y., Yuan H. S. (2013). *Ganodermamutabile* sp. nov. from southwestern China based on morphological and molecular data. Mycological Progress.

[B7614749] Chang T. T., Chen T. (1984). *Ganodermaformosanum* sp. nov on formosan sweet gum in Taiwan. Transactions of the British Mycological Society.

[B7840701] Chao Yun (2013). Taxonomy and Phylogeny of *Ganoderma* in China.

[B7614796] Coetzee M. P.A., Marincowitz S., Muthelo V. G., Wingfield M. J. (2015). *Ganoderma* species, including new taxa associated with root rot of the iconic *Jacarandamimosifolia* in Pretoria, South Africa. IMA Fungus.

[B7614832] Costa-Rezende D. H., Robledo G. L., Góes-Neto A., Reck M. A., Crespo E., Drechsler-Santos E. R. (2017). Morphological reassessment and molecular phylogenetic analyses of *Amauroderma* s.lat. raised new perspectives in the generic classification of the Ganodermataceae family. Persoonia.

[B7614805] Costa-Rezende D. H., Robledo G. L., Drechsler-Santos E. R., Glen M., Gates G., Bonz B. R.M., Popof O. F., Crespo E., Góes-Neto A (2020). Taxonomy and phylogeny of polypores with ganodermatoid basidiospores (Ganodermataceae). Mycological Progress.

[B7623211] Crous P. W., Wingfield M. J., Roux J. J. Le, Richardson D. M., Strasberg D., Shivas R. G., Alvarado P., Edwards J., Moreno G., Sharma R., Sonawane M. S., Tan Y. P., Altés A., Barasubiye T., Barnes C. W., Blanchette R. A., Boertmann D., Bogo A., Carlavilla J. R., Cheewangkoon R., Daniel R., de Beer Z. W., Yáñez-Morales M. de Jesús, Duong T. A., Fernández-Vicente J., Geering A. D.W., Guest D. I., Held B. W., Heykoop M., Hubka V., Ismail A. M., Kajale S. C., Khemmuk W., Kolařík M., Kurli R., Lebeuf R., Lévesque C. A., Lombard L., Magista D., Manjón J. L., Marincowitz S., Mohedano J. M., Nováková A., Oberlies N. H., Otto E. C., Paguigan N. D., Pascoe I. G., Pérez-Butrón J. L., Perrone G., Rahi P., Raja H. A., Rintoul T., Sanhueza R. M.V., Scarlett K., Shouche Y. S., Shuttleworth L. A., Taylor P. W.J., Thorn R. G., Vawdrey L. L., Solano-Vidal R., Voitk A., Wong P. T.W., Wood A. R., Zamora J. C., Groenewald J. Z. (2015). Fungal planet description sheets: 371–399. Persoonia.

[B7623012] Crous P. W., Wingfield M. J., Richardson D. M., Leroux J. J., Strasberg D., Edwards J., Roets F., Hubka V., Taylor P. W.J., Heykoop M., Martín M. P., Moreno G., Sutton D. A., Wiederhold N. P., Barnes C. W., Carlavilla J. R., Gené J., Giraldo A., Guarnaccia V., Guarro J., Hernández-Restrepo M., Kolařík M., Manjón J. L., Pascoe I. G., Popov E. S., Sandoval-Denis M., Woudenberg J. H.C., Acharya K., Alexandrova A. V., Alvarado P., Barbosa R. N., Baseia I. G., Blanchette R. A., Boekhout T., Burgess T. I., Cano-Lira J. F., Čmoková A., Dimitrov R. A., Dyakov M. Yu., Dueñas M., Dutta A. K., Esteve-Raventós F., Fedosova A. G., Fournier J., Gamboa P., Gouliamova D. E., Grebenc T., Groenewald M., Hanse B., Hardy G. E.ST.J., Held B. W., Jurjević Ž, Kaewgrajang T., Latha K. P.D., Lombard L., Luangsa-ard J. J., Lysková P., Mallátová N., Manimohan P., Miller A. N., Mirabolfathy M., Morozova O. V., Obodai M., Oliveira N. T., Ordóñez M. E., Otto E. C., Paloi S., Peterson S. W., Phosri C., Roux J., Salazar W. A., Sánchez A., Sarria G. A., Shin H. -D., Silva B. D.B., Silva G. A., Smith M. TH., Souza-Motta C. M., Stchigel A. M., Stoilova-Disheva M. M., Sulzbacher M. A., Telleria M. T., Toapanta C., Traba J. M., Valenzuela-Lopez N., Watling R., Groenewald J. Z. (2016). Fungal planet description sheets: 400–468. Persoonia.

[B7623123] Crous P. W., Wingfield M. J., Burgess T. I., Hardy G. E.ST.J., Barber P. A., Alvarado P., Barnes C. W., Buchanan P. K., Heykoop M., Moreno G., Thangavel R., Van der spuy S., Barili A., Barrett S., Cacciola S. O., Cano-Lira J. F., Crane C., Decock C., Gibertoni T. B., Guarro J., Guevara-Suarez M., Hubka V., Kolařík M., Lira C. R.S., Ordoñez M. E., Padamsee M., Ryvarden L., Soares A. M., Stchigel A. M., Sutton D. A., Vizzini A., Weir B. S., Acharya K., Aloi F., Baseia I. G., Blanchette R. A., Bordallo J. J., Bratek Z., Butler T., Cano-Canals J., Carlavilla J. R., Chander J., Cheewangkoon R., Cruz R. H.S.F., Da silva M., Dutta A. K., Ercole E., Escobio V., Esteve-Raventós F., Flores J. A., Gené J., Góis J. S., Haines L., Held B. W., Horta jung M., Hosaka K., Jung T., Jurjević Ž., Kautman V., Kautmanova I., Kiyashko A. A., Kozanek M., Kubátová A., Lafourcade M., La spada F., Latha K. P.D., Madrid H., Malysheva E. F., Manimohan P., Manjón J. L., Martín M. P., Mata M., Merényi Z., Morte A., Nagy I., Normand A. -C., Paloi S., Pattison N., Pawłowska J., Pereira O. L., Petterson M. E., Picillo B., Raj K. N.A. (2017). Fungal planet description sheets: 558–624. Persoonia.

[B7614843] Cui B. K., Li H. J., Zhou J. L., Song J., Si J., Yang Z. L., Dai Y. C. (2019). Species diversity, taxonomy and phylogeny of Polyporaceae (Basidiomycota) in China. Fungal Diversity.

[B7614855] Dai Yu. Cheng, Yang Zhu. Liang, Cui Bao. Kai, Yu Chang. Jun, Zhou Li. Wei (2009). Species diversity and utilization of medicinal mushrooms and fungi in China (Review). International Journal of Medicinal Mushrooms.

[B7614865] Demoulin Vincent (2010). Why conservation of the name *Boletusapplanatus* should be rejected. Taxon.

[B7614874] Ding Shun. Ping, Hu Hong. Li, Gu Ji. Dong (2020). Diversity, abundance, and distribution of wood-decay fungi in major parks of Hong Kong. Forests.

[B7614896] Douanla-Meli Clovis, Langer Ewald (2009). *Ganodermacarocalcareus* sp. nov., with crumbly-friable context parasite to saprobe on *Anthocleistanobilis* and its phylogenetic relationship in *G.resinaceum* group. Mycological Progress.

[B7839943] Du Q., Cao Y., Liu C., Du Q. (2021). The Lingzhi Mushroom Genome, Compendium of Plant Genomes.

[B7614905] Fryssouli Vassiliki, Zervakis Georgios, Polemis Elias, Typas Milton A. (2020). A global meta-analysis of ITS rDNA sequences from material belonging to the genus *Ganoderma* (Basidiomycota, Polyporales) including new data from selected taxa. MycoKeys.

[B7614928] Glen M., Yuskianti V., Puspitasari D., Francis A., Agustini L., Rimbawanto A., Indrayadi H., Gafur A., Mohammed C. L. (2014). Identification of Basidiomycete fungi in Indonesian hardwood plantations by DNA barcoding. Forest Pathology.

[B7614942] Gottlieb Alexandra M., Ferrer Esther, Wright Jorge E. (2000). rDNA analyses as an aid to the taxonomy of species of *Ganoderma*. Mycological Research.

[B7614951] Gunnels Tess, Creswell Matthew, McFerrin Janis, Whittall Justen B. (2020). The ITS region provides a reliable DNA barcode for identifying reishi/lingzhi (*Ganoderma*) from herbal supplements. PLoS One.

[B7614983] Hall T. A. (1999). BioEdit: A user-friendly biological sequence alignment editor andanalysis program for Windows 95/98/NT. Nucleic Acids Symposium Series.

[B7615084] Hapuarachchi K. K., Wen T. C., Deng C. Y., Kang J. C., Hyde K. D. (2015). Mycosphere essays 1: Taxonomic confusion in the *Ganodermalucidum* species complex. Mycosphere.

[B7615105] Hapuarachchi K. K., Wen T. C., Jeewon R., Wu X. L., Kang J. C (2016). Mycosphere essays 15: *Ganodermalucidum* - are the beneficial medical properties substantiated?. Mycosphere.

[B7615094] Hapuarachchi K. K., Wen T. C., Jeewon R., Wu X. L., Kang J. C., Hyde K. D. (2016). Mycosphere essays 7. *Ganodermalucidum* - are the beneficial anti-cancer properties substantiated?. Mycosphere.

[B7615115] Hapuarachchi K. K., Cheng C. R., Wen T. C., Jeewon R., Kakumyan P. (2017). Mycosphere essays 20: Therapeutic potential of *Ganoderma* species: Insights into its use as traditional medicine. Mycosphere.

[B7615047] Hapuarachchi K. K., Karunarathna S. C., Phengsintham P., Kakumyan P., Hyde K. D., Wen T. C. (2018). *Amauroderma* (Ganodermataceae, Polyporales) - bioactive compounds, beneficial properties and two new records from Laos. Asian Journal of Mycology.

[B7615021] Hapuarachchi K. K., Elkhateeb W. A., Karunarathna S. C., Cheng C. R., Bandara A. R., Kakumyan P., Hyde K. D., Daba G. M., Wen T. C. (2018). Current status of global *Ganoderma* cultivation, products, industry and market. Mycosphere.

[B7615070] Hapuarachchi K. K., Karunarathna S. C., Rasp&eacute O., De Silva K. H.W.L., Thawthong A., Wu X. L., Kakumyan P., Hyde K. D., Wen T. C. (2018). High diversity of *Ganoderma* and *Amauroderma* (Ganodermataceae, Polyporales) in Hainan Island, China. Mycosphere.

[B7615035] Hapuarachchi K. K., Karunarathna S. C., McKenzie E. H.C., Wu X. L., Kakumyan P., Hyde K. D., Wen T. C. (2019). High phenotypic plasticity of *Ganodermasinense* (Ganodermataceae, Polyporales) in China. Asian Journal of Mycology.

[B7615058] Hapuarachchi K. K., Karunarathna S. C., Phengsintham P., Yang H. D., Kakumyan P., Hyde K. D., Wen T. C. (2019). Ganodermataceae (Polyporales): Diversity in Greater Mekong Subregion countries (China, Laos, Myanmar, Thailand and Vietnam). Mycosphere.

[B7838370] He Jun, Luo Zong-Long, Tang Song-Ming, Li Yong-Jun, Li Shu-Hong, Su Hong-Yan (2021). Phylogenetic analyses and morphological characters reveal two new species of *Ganoderma* from Yunnan province, China. MycoKeys.

[B7616665] He Mao. Qiang, Zhao Rui. Lin, Hyde Kevin. D., Begerow Dominik, Kemler Martin, Yurkov Andrey, McKenzie Eric. H. C., Raspé Olivier, Kakishima Makoto, Sánchez-Ramírez Santiago, Vellinga Else. C., Halling Roy, Papp Viktor, Zmitrovich Ivan. V., Buyck Bart, Ertz Damien, Wijayawardene Nalin. N., Cui Bao. Kai, Schoutteten Nathan, Liu Xin. Zhan, Li Tai. Hui, Yao Yi. Jian, Zhu Xin. Yu, Liu An. Qi, Li Guo. Jie, Zhang Ming. Zhe, Ling Zhi. Lin, Cao Bin, Antonín Vladimír, Boekhout Teun, da Silva Bianca Denise Barbosa, De Crop Eske, Decock Cony, Dima Bálint, Dutta Arun. Kumar, Fell Jack. W., Geml József, Ghobad-Nejhad Masoomeh, Giachini Admir. J., Gibertoni Tatiana. B., Gorjón Sergio. P., Haelewaters Danny, He Shuang. Hui, Hodkinson Brendan. P., Horak Egon, Hoshino Tamotsu, Justo Alfredo, Lim Young. Woon, Menolli Nelson, Mešić Armin, Moncalvo Jean. Marc, Mueller Gregory. M., Nagy László. G., Nilsson R. Henrik, Noordeloos Machiel, Nuytinck Jorinde, Orihara Takamichi, Ratchadawan Cheewangkoon, Rajchenberg Mario, Silva-Filho Alexandre. G. S., Sulzbacher Marcelo. Aloisio, Tkalčec Zdenko, Valenzuela Ricardo, Verbeken Annemieke, Vizzini Alfredo, Wartchow Felipe, Wei Tie. Zheng, Weiß Michael, Zhao Chang. Lin, Kirk Paul. M. (2019). Notes, outline and divergence times of Basidiomycota. Fungal Diversity.

[B7838173] He Mao Qiang, Zhao Rui Lin, Liu Dong Mei, Denchev Teodor T., Begerow Dominik, Yurkov Andrey, Kemler Martin, Millanes Ana M., Wedin Mats, McTaggart A. R., Shivas Roger G., Buyck Bart, Chen Jie, Vizzini Alfredo, Papp Viktor, Zmitrovich Ivan V., Davoodian Naveed, Hyde Kevin D. (2022). Species diversity of Basidiomycota. Fungal Diversity.

[B7840663] He Shao Chang, Yu Hui Fang (1989). The Family Ganodermataceae from Guizhou province of China. Acta Mycologica Sinca.

[B7615200] Hushiarian Roozbeh, Yusof Nor Azah, Dutse Sabo Wada (2013). Detection and control of *Ganodermaboninense*: strategies and perspectives. Springer Plus.

[B7615209] Imazeki R. (1939). Studies on *Ganoderma* of Nippon. Bulletin of the Tokyo Science Museum.

[B7615240] Jargalmaa S., Eimes J. A., Park M. S., Park J. Y., Oh S. Y., Lim Y. W. (2017). Taxonomic evaluation of selected *Ganoderma* species and database sequence validation. PeerJ.

[B7615251] Jayasiri Subashini. C., Hyde Kevin. D., Ariyawansa Hiran. A., Bhat Jayarama, Buyck Bart, Cai Lei, Dai Yu. Cheng, Abd-Elsalam Kamel. A., Ertz Damien, Hidayat Iman, Jeewon Rajesh, Jones E. B. Gareth, Bahkali Ali. H., Karunarathna Samantha. C., Liu Jian. Kui, Luangsa-ard J. Jennifer, Lumbsch H. Thorsten, Maharachchikumbura Sajeewa. S. N., McKenzie Eric. H. C., Moncalvo Jean. Marc, Ghobad-Nejhad Masoomeh, Nilsson Henrik, Pang Ka. Lai, Pereira Olinto. L., Phillips Alan J. L., Raspé Olivier, Rollins Adam. W., Romero Andrea. I., Etayo Javier, Selçuk Faruk, Stephenson Steven. L., Suetrong Satinee, Taylor Joanne. E., Tsui Clement K. M., Vizzini Alfredo, Abdel-Wahab Mohamed. A., Wen Ting. Chi, Boonmee Saranyaphat, Dai Dong. Qin, Daranagama Dinushani. A., Dissanayake Asha. J., Ekanayaka Anusha. H., Fryar S. C., Hongsanan Sinang, Jayawardena Ruvishika. S., Li Wen. Jing, Perera Rekhani. H., Phookamsak R., de Silva Nimali. I., Thambugala Kasun. M., Tian Qing, Wijayawardene Nalin. N., Zhao Rui. Lin, Zhao Qi, Kang Ji. Chuan, Promputtha Itthayakorn (2015). The faces of fungi database: fungal names linked with morphology, phylogeny and human impacts. Fungal Diversity.

[B7615312] Jiang Nan, Hu Shuang, Peng Bing, Li Zhen. Hao, Yuan Xiao. Hui, Xiao Shi. Jun, Fu Yong. Ping (2021). Genome of *Ganoderma* species provides insights into the evolution, conifers substrate utilization, and terpene synthesis for *Ganodermatsugae*. Frontiers in Microbiology.

[B7615324] Justo Alfredo, Miettinen Otto, Floudas Dimitrios, Ortiz-Santana Beatriz, Sjökvist Elisabet, Lindner Daniel, Nakasone Karen, Niemelä Tuomo, Larsson Karl. Henrik, Ryvarden Leif, Hibbett David. S. (2017). A revised family-level classification of the Polyporales (Basidiomycota). Fungal Biology.

[B7615340] Kadowaki Kohmei, Leschen Richard. A.B., Beggs Jacqueline. R. (2011). No evidence for a *Ganoderma* spore dispersal mutualism in an obligate spore-feeding beetle *Zearagytodesmaculifer*. Fungal Biology.

[B7616805] Karsten P. A. (1881). Enumeralio boletinearum et polyporearum fennicarum, systemate novo dispositarum. Revue de Mycologie.

[B7615359] Katoh K., Standley D. M. (2013). MAFFT multiple sequence alignment software version 7: Improvements in performance and usability. Molecular Biology and Evolution.

[B7615368] Keypour Somayeh, Riahi Hossein, Asef Mohammad. Reza, Abdollahzadeh Jafar, borhani Ali, Safaie Naser (2020). The true nature of *Ganoderma* in Iran: Taxonomy based on ITS and mtSSU rDNA. Forest Pathology.

[B7615379] Kinge T. R., Mih A. M., Coetzee M. P.A. (2012). Phylogenetic relationships among species of *Ganoderma* (Ganodermataceae, Basidiomycota) from Cameroon. Australian Journal of Botany.

[B7615404] Kornerup A, Wanscher J. H (1978). Methuen Handbook of Colour.

[B7615412] Kües Ursula, Nelson David R., Liu Chang, Yu Guo. Jun, Zhang Jian. Hui, Li Jian. Qin, Wang Xin. Cun, Sun Hui (2015). Genome analysis of medicinal *Ganoderma* spp. with plant-pathogenic and saprotrophic life-styles. Phytochemistry.

[B7838602] Lanfear R., Frandsen P. B., Wright A. M. (2017). PartitionFinder 2: New methods for selecting partitioned models of evolution for molecular and morphological phylogenetic analyses. Molecular Biology and Evolution.

[B7615425] Larget B., Simon D. L. (1999). Markov chain Monte Carlo algorithms for the Bayesian analysis of phylogenetic trees. Molecular Biology and Evolution.

[B7615434] Lee S. S., Chang Y. S. (2016). *Ganoderma* - Jekyll and Hyde mushrooms. Agriculture Science Journal.

[B7836014] Leonardo-Silva Lucas, Cotrim Carlos Filipe Camilo, Xavier-Santos Solange (2022). Furtadomyces nom. nov. (Ganodermataceae, Basidiomycota) with description of F. sumptuosus, a new species of ganodermatoid fungi from Brazil. Mycological Progress.

[B7615443] Li Li Feng, Liu Hong Bing, Zhang Quan Wei, Li Zhi Peng, Wong Tin Long, Fung Hau Yee, Zhang Ji Xia, Bai Su Ping, Lu Ai Ping, Han Quan Bin (2018). Comprehensive comparison of polysaccharides from *Ganodermalucidum* and *G.sinense*: chemical, antitumor, immunomodulating and gut-microbiota modulatory properties. Scientific Reports.

[B7615495] Lin Z. B, Yang B. X (2019). *Ganoderma* and health: Biology, chemistry and industry.

[B7622992] Li Tai. Hui, Hu Hui. Ping, Deng Wang. Qiu, Wu Sheng. Hua, Wang Dong. Mei, Tsering Tamdrin (2015). *Ganodermaleucocontextum*, a new member of the *G.lucidum* complex from southwestern China. Mycoscience.

[B7615503] Liu HONG, Guo LI. JIE, Li SU. LING, Fan LI (2019). *Ganodermashanxiense*, a new species from northern China based on morphological and molecular evidence. Phytotaxa.

[B7615512] Liu Y. J., Whelen S., Hall B. D. (1999). Phylogenetic relationships among ascomycetes: evidence from an RNA polymerse II subunit. Molecular Biology and Evolution.

[B7615521] López-Peña Damian, Samaniego-Rubiano Crystal, Morales-Estrada Idaly, Gutierrez Aldo, Gaitán-Hernández Rigoberto, Esqueda Martín (2019). Morphological characteristics of wild and cultivated *Ganodermasubincrustatum* from Sonora, Mexico. Scientia Fungorum.

[B7615532] Loyd A. L., Barnes C. W., Held B. W., Schink M. J., Smith M. E., Smith J. A., Blanchette R. A. (2018). Elucidating "lucidum": Distinguishing the diverse laccate *Ganoderma* species of the United States. PLOS One.

[B7615565] Luangharn Thatsanee, Karunarathna Samantha. C., Mortimer Peter. E., Hyde Kevin. D., Xu Jian. Chu (2019). Additions to the knowledge of *Ganoderma* in Thailand: *Ganodermacasuarinicola*, a new record; and *Ganodermathailandicum* sp. nov.. MycoKeys.

[B7615554] Luangharn T., Karunarathna S. C., Mortimer P. E., Hyde K. D., Thongklang N., Xu J. C. (2019). A new record of *Ganodermatropicum* (Basidiomycota, Polyporales) for Thailand and first assessment of optimum conditions for mycelia production. MycoKeys.

[B7615586] Mafia M. I., Aminuzzaman F. M., Chowdhury Mohammad. Salahuddin. Mahmood, Tanni Jannatul. Ferdous (2020). Occurrence, diversity and morphology of poroid wood decay by *Ganoderma* spp. from tropical moist deciduous forest region of Bangladesh. Journal of Agriculture and Natural Resources.

[B7615595] Midot F, Lau S. Y. L, Wong W. C, Tung H. J, Yap M. L, Lo M. L, Jee M. S, Dom S. P, Melling L (2019). Genetic dversity and demographic history of *Ganodermaboninense* in oil palm plantations of Sarawak, Malaysia inferred from ITS regions. Microorganisms.

[B7634275] Miller M. A., Pfeiffer W, Schwartz T (2010). Creating the CIPRES Science Gateway for inference of large phylogenetic trees.

[B7615618] Mohd Shukri. I, Izzuddin M. A, Mohd Hefni. R, Idris A. S (2020). Geostatistics of oil palm trees affected by *Ganoderma* disease in low and high planting density. IOP Conference Series: Earth and Environmental Science.

[B7874175] Moncalvo Jean-Marc, Buchanan Peter K. (2008). Molecular evidence for long distance dispersal across the Southern Hemisphere in the Ganoderma
applanatum-australe species complex (Basidiomycota). Mycological Research.

[B7615627] Moncalvo J. M., Ryvarden L. (1997). A nomenclatural study of the Ganodermataceae Donk. Synopsis Fungorum.

[B7615636] Murrill William Alphonso (1902). The Polyporaceae of North America. I. The genus *Ganoderma*. Bulletin of the Torrey Botanical CLub.

[B7615645] Náplavová Kateřina, Beck Terézia, Pristaš Peter, Gáperová Svetlana, Šebesta Martin, Piknová Mária, Gáper Ján (2020). Molecular data reveal unrecognized diversity in the European *Ganodermaresinaceum*. Forests.

[B7615698] Papp V. (2016). The first validly published laccate *Ganoderma* species from East Asia: *G.dimidiatum* comb. nov., the correct name for *G.japonicum*. Studia Botanica Hungarica.

[B7615707] Pegler D. N., Young T. W.K. (1973). Basidiospore form in the British species of *Ganoderma* Karst. Kew Bulletin.

[B7615716] Pinruan Umpava, Rungjindamai Nattawut, Choeyklin Rattaket, Lumyong Saisamorn, Hyde Kevin. D., Jones E. B. Gareth (2010). Occurrence and diversity of basidiomycetous endophytes from the oil palm, *Elaeisguineensis* in Thailand. Fungal Diversity.

[B7615727] Rambaut A (2012). FigTree version 1.4.0. http://tree.bio.ed.ac.uk/software/fgtree/.

[B7615758] Rambaut A, Suchard M. A, Xie D, Drummond A. J (2013). Tracer v 1.6. http://tree.bio.ed.ac.uk/software/tracer/.

[B7615766] Rees R. W., Flood J., Hasan Y., Potter U., Cooper R. M. (2009). Basal stem rot of oil palm (*Elaeisguineensis*); mode of root infection and lower stem invasion by *Ganodermaboninense*. Plant Pathology.

[B7615776] Rees R. W., Flood J., Hasan Y., Wills M. A., Cooper R. M. (2012). *Ganodermaboninense* basidiospores in oil palm plantations: evaluation of their possible role in stem rots of *Elaeisguineensis*. Plant Pathology.

[B7838404] Rehner Stephen A., Buckley Ellen (2017). A Beauveria phylogeny inferred from nuclear ITS and EF1-α sequences: evidence for cryptic diversification and links to *Cordycepsteleomorphs*. Mycologia.

[B7615786] Richter Christian, Wittstein Kathrin, Kirk Paul. M., Stadler Marc (2014). An assessment of the taxonomy and chemotaxonomy of *Ganoderma*. Fungal Diversity.

[B7838033] Ryvarden Leif (2000). Studies in Neotropical Polypores 2: A Preliminary Key to Neotropical Species of Ganoderma with a Laccate Pileus. Mycologia.

[B7616553] Sadyś M, Skjøth C. A, Kennedy R (2014). Back-trajectories show export of airborne fungal spores (*Ganoderma* sp.) from forests to agricultural and urban areas in England. Atmospheric Environment.

[B7615819] Seo G. S., Kitamot Y. (1998). Morphological features and morphogenesis in the *Ganodermalucidum* complex. Japanese Society of Mushroom Science and Biotechnology.

[B7615828] Shen Shan, Liu Shi. Liang, Jiang Ji. Hang, Zhou Li. Wei (2021). Addressing widespread misidentifications of traditional medicinal mushrooms in *Sanghuangporus* (Basidiomycota) through ITS barcoding and designation of reference sequences.. IMA fungus.

[B7615855] Smith Brendan. J., Sivasithamparam K. (2003). Morphological studies of *Ganoderma* (Ganodermataceae) from the Australasian and Pacific regions. Australian Systematic Botany.

[B7615874] Song J., Xing J. H., Decock C., He X. L., Cui B. K. (2016). Molecular phylogeny and morphology reveal a new species of *Amauroderma* (Basidiomycota) from China. Phytotaxa.

[B7615884] Stamatakis A. (2014). RAxML version 8: a tool for phylogenetic analysis and post-analysis of large phylogenies. Bioinformatics.

[B7615893] Steyaert R. L. (1980). Study of some *Ganoderma* species. Bulletin du Jardin Botanique National de Belgique.

[B7615911] Sun Y. F, Costa-Rezende D. H, Xing J. H, Zhou J. L, Zhang B, Gibertoni T. B, Gates G, Glen M, Dai Y. C, Cui B. K (2020). Multi-gene phylogeny and taxonomy of *Amauroderma* s. lat. (Ganodermataceae). Persoonia.

[B7615926] Szedlay Z., Jakucs E., Boldizsar I., Boka A. (1999). Basidiocarp and mycelium morphology of *Ganodermalucidum* Karst. strains isolated in hungary. Acta Microbiologica et Immunologica Hungarica.

[B7615935] Tchotet-Tchoumi James Michel, Coetzee Martin Petrus Albertus, Rajchenberg Mario, Roux Jolanda (2019). Taxonomy and species diversity of *Ganoderma* species in the Garden Route National Park of South Africa inferred from morphology and multilocus phylogenies. Mycologia.

[B7615944] Torres-Torres Mabel Gisela, Guzmán-Dávalos Laura (2012). The morphology of *Ganoderma* species with a laccate surface. Mycotaxon.

[B7615953] Tuno Nobuko (1999). Insect feeding on spores of a bracket fungus, *Elfvingiaapplanata* (Pers.) Karst. (Ganodermataceae, Aphyllophorales). Ecological Research.

[B7615962] Vaidya Gaurav, Lohman David. J., Meier Rudolf (2011). SequenceMatrix: concatenation software for the fast assembly of multi-gene datasets with character set and codon information. Cladistics.

[B7615971] Vilgalys R, Hester M (1990). Rapid genetic identification and mapping of enzymatically amplified ribosomal DNA from several *Cryptococcus* species. Journal of Bacteriology.

[B7623104] Vu D., Groenewald M., de Vries M., Gehrmann T., Stielow B., Eberhardt U., Al-Hatmi A., Groenewald J. Z., Cardinali G., Houbraken J., Boekhout T., Crous P. W., Robert V., Verkley G. J.M. (2019). Large-scale generation and analysis of filamentous fungal DNA barcodes boosts coverage for kingdom fungi and reveals thresholds for fungal species and higher taxon delimitation. Studies in Mycology.

[B7615980] Wang Guang. Yuan, Xu L. L, Yu Hao, Gao Jie, Guo Li. Zhong (2019). Systematic analysis of the lysine succinylome in the model medicinal mushroom *Ganodermalucidum*. BMC Genomics.

[B7840709] Wang Xing Cun (2012). Phylogenetic Study on Ganodermataceae Donk.

[B7615999] Welti Stéphane, Moreau Pierre. Arthur, Decock Cony, Danel Cécile, Duhal Nathalie, Favel Anne, Courtecuisse Régis (2015). Oxygenated lanostane-type triterpenes profiling in laccate *Ganoderma* chemotaxonomy. Mycological Progress.

[B7616562] White T. J., Bruns T., Lee S., Taylor J., Innis M. A., Gelfand D. H., Sninsky J. J., White T. J. (1990). PCR Protocols: a guide to methods and applications.

[B7616079] Wong C. L., Bong J. F.C., Idris A. S. (2012). *Ganoderma* species associated with basal stem rot disease of oil palm. American Journal of Applied Sciences.

[B7616089] Wu Fang, Zhou Li. Wei, Yang Zhu. Liang, Bau Tolgor, Li Tai. Hui, Dai Yu. Cheng (2019). Resource diversity of Chinese macrofungi: edible, medicinal and poisonous species. Fungal Diversity.

[B7616109] Wu SHENG. HUA, Chern CHI. LIANG, Wei CHIA. LING, Chen YU. PING, Akiba MITSUTERU, Hattori TSUTOMU (2020). *Ganodermabambusicola* sp. nov. (Polyporales, Basidiomycota) from southern Asia. Phytotaxa.

[B7616120] Wu X. L., Dai Y. C., Lin Y. H. (2004). Study on the Ganodermataceae of China Ⅲ. Guizhou Sciense.

[B7840693] Wu X. L., Dai Y. C. (2005). Coloured illustrations of Ganodermataceae of China.

[B7616170] Xing JIA. HUI, Song JIE, Decock CONY, Cui BAO. KAI (2016). Morphological characters and phylogenetic analysis reveal a new species within the *Ganodermalucidum* complex from South Africa. Phytotaxa.

[B7616189] Xing Jia. Hui, Sun Yi. Fei, Han Yu. Li, Cui Bao. Kai, Dai Yu. Cheng (2018). Morphological and molecular identification of two new *Ganoderma* species on *Casuarinaequisetifolia* from China. MycoKeys.

[B7634446] Xing J. H (2019). Species diversity, taxonomy and phylogeny of *Ganoderma*.

[B7616199] Yamashita Satoshi, Hirose Dai (2016). Phylogenetic analysis of *Ganodermaaustrale* complex in a Bornean tropical rainforest and implications for mechanism of coexistence of various phylogenetic types. Fungal Ecology.

[B7616208] Yang Z. L., Feng B. (2013). What is the Chinese “Lingzhi”? - a taxonomic mini-review. Mycology.

[B7616274] Yao Y. J., Wang X. C., Wang B. (2013). Epitypification of *Ganodermasichuanense* J.D. Zhao & X.Q. Zhang (Ganodermataceae). Taxon.

[B7616257] Ye L., Karunarathna S. C., Mortimer P. E., Li H., Qiu M. H., Peng X. R., Luangharn T., Li Y. J., I Prom-, Hyde K. D., Xu J. C. (2019). *Ganodermaweixiensis* (Polyporaceae, Basidiomycota), a new member of the *G.lucidum* complex from Yunnan Province, China. Phytotaxa.

[B7840673] Zhang Xiao Qing (1997). Four new records of Ganodermataceae from China. Mycosystema.

[B7840629] Zhao J. D. (1989). The Ganodermataceae in China. Bibliotheca Mycologica.

[B7616314] Zhao J. D, Zhang X. Q (2000). Flora fungorum cinicorum.

[B7616340] Zhao Rui. Lin, Li Guo. Jie, Sánchez-Ramírez Santiago, Stata Matt, Yang Zhu. Liang, Wu Gang, Dai Yu. Cheng, He Shuang. Hui, Cui Bao. Kai, Zhou Jun. Liang, Wu Fang, He Mao. Qiang, Moncalvo Jean. Marc, Hyde Kevin. D. (2017). A six-gene phylogenetic overview of Basidiomycota and allied phyla with estimated divergence times of higher taxa and a phyloproteomics perspective. Fungal Diversity.

[B7616359] Zhou Li. Wei, Cao Yun, Wu Sheng. Hua, Vlasák Josef, Li De. Wei, Li Meng. Jie, Dai Yu. Cheng (2015). Global diversity of the *Ganodermalucidum* complex (Ganodermataceae, Polyporales) inferred from morphology and multilocus phylogeny. Phytochemistry.

[B7616371] Zhou Xuan. Wei, Liu Yan, Guo Meng. Yuan, Su Ka. Qi, Zhang Yong. Ming (2016). Species clarification of the widely cultivated *Ganoderma* in China based on rDNA and FIP gene sequence analysis. International Journal of Agriculture and Biology.

